# Atezolizumab plus bevacizumab treatment for unresectable hepatocellular carcinoma progressing after molecular targeted therapy: A multicenter prospective observational study

**DOI:** 10.1097/MD.0000000000030871

**Published:** 2022-10-07

**Authors:** Rie Sugimoto, Takeaki Satoh, Akihiro Ueda, Takeshi Senju, Yuki Tanaka, Shinsaku Yamashita, Toshimasa Koyanagi, Tomoyuki Kurashige, Nobito Higuchi, Tsukasa Nakamura, Masatake Tanaka, Yuuki Azuma, Akari Ohno, Aritsune Ooho, Mari Ooe, Taiji Mutsuki, Koutarou Uchimura, Masami Kuniyoshi, Seiya Tada, Yoshifusa Aratake, Tsuyoshi Yoshimoto, Naoki Yamashita, Shigeru Harada, Makoto Nakamuta, Kenta Motomura, Motoyuki Kohjima

**Affiliations:** a Department of Hepato-Biliary-Pancreatology, National Hospital Organization Kyushu Cancer Center, Fukuoka City, Fukuoka, Japan; b Department of Center for Liver Disease, Kokura Medical Center, Kitakyushu City, Fukuoka, Japan; c Department of Internal Medicine, Fukuoka City Hospital, Fukuoka City, Fukuoka, Japan; d Department of Medicine and Bioregulatory Science, Graduate School of Medical Sciences, Kyushu University, Fukuoka City, Fukuoka, Japan; e Department of Hepatology, Steel Memorial Yawata Hospital, Kitakyushu City, Fukuoka, Japan; f Social Insurance Nakabaru Hospital, Kasuya, Fukuoka, Japan; g Department of Gastroenterology, Kyushu Rosai Hospital, Kitakyushu City, Fukuoka, Japan; h Department of Gastroenterology and Hepatology, Fukuokahigashi Medical Center, Koga City, Fukuoka, Japan; i Department of Gastroenterology, National Hospital Organization Kyushu Medical Center, Fukuoka City, Fukuoka, Japan; j Department of Gastroenterology, Chihaya Hospital, Fukuoka, Japan; k Department of Hepatology, Aso Iizuka Hospital, Iizuka City, Fukuoka, Japan.

**Keywords:** atezolizumab plus bevacizumab, hepatocellular carcinoma; RECIST, mRECIST, MTA pretreatment

## Abstract

To evaluate the efficacy of atezolizumab plus bevacizumab treatment in patients with hepatocellular carcinoma (HCC) previously treated with molecular targeted agents (MTAs).

Thirty-one patients treated with atezolizumab plus bevacizumab for unresectable HCC and previously treated with MTAs were enrolled in this study.

The treatment lines ranged from second to sixth lines. The treatment effect on HCC differed from that during first-line treatment. The treatment effect was determined using the Response Evaluation Criteria in Solid Tumors (RECIST) and modified RECIST. The treatment response was different for each MTA immediately prior to atezolizumab + bevacizumab treatment. Tumors treated with lenvatinib followed by atezolizumab + bevacizumab showed rapid growth for a short period of time followed by shrinkage. However, patients who received ramucirumab, sorafenib, and regorafenib did not show such changes. This was likely because of differences in the mechanism of action of the MTA administered immediately beforehand. The side-effect profile differed from that observed in the IMbrave150 phase 3 study of atezolizumab plus bevacizumab, which showed more adverse events related to hepatic reserve.

Patients treated with the combination of atezolizumab and bevacizumab after lenvatinib therapy may experience rapid tumor growth and subsequent shrinkage.

## 1. Introduction

Several molecular targeted agents (MTAs) have been developed for the treatment of unresectable hepatocellular carcinoma (HCC), including sorafenib, lenvatinib, regorafenib, ramucirumab, and cabozantinib.^[1–5]^ Atezolizumab plus bevacizumab is the first combination therapy to incorporate a checkpoint inhibitor and MTA for HCC and showed significantly better overall survival (OS) and progression-free survival (PFS) than sorafenib in the treatment of unresectable HCC in a phase 3 trial, IMbrave150.^[6]^ However, this study was conducted in patients with no previous therapy,^[6]^ and there is no evidence that the same results can be achieved in patients who have already received MTA treatment. To examine this further, we conducted a multicenter observational study to determine the early outcomes and changes in liver reserve following atezolizumab plus bevacizumab treatment in pretreated unresectable HCC. We investigated the efficacy and safety of late-line atezolizumab plus bevacizumab treatment in the real world by examining the clinical characteristics of patients and validating the treatment results. This study was approved by the ethics committee of Kyushu Cancer Center (2018–16) and performed in compliance with the 1975 Declaration of Helsinki.

## 2. Materials and Methods

### 2.1. Patients

Three hundred twenty-four patients who received MTA treatment for unresectable HCC at our hospital and related facilities from 2018 to December 2022 were enrolled in this prospective observational study (Iizuka Hospital, n = 94; Fukuoka City Hospital, n = 36; Fukuoka Higashi Medical Center, n = 14; Kyushu Cancer Center, n = 84; Kyushu Medical Center, n = 26; Kokura Medical Center, n = 29; Kyushu University Hospital, n = 13; Nakabaru Hospital, n = 1; Steel Memorial Yawata Hospital, n = 24; Kyushu Rosai Hospital, n = 2; Chihaya Hospital, n = 1). Thirty-one patients with advanced unresectable HCC previously treated with MTAs, who received at least 1 MTA followed by atezolizumab plus bevacizumab between September 2020 and January 2022, were enrolled in this study. To prevent selection bias, all patients who gave consent were enrolled in this observational study. All eligible patients consecutively consented to the trial, with no patients refusing consent. We examined the records of these patients and collected the relevant data. Clinical characteristics, prognostic factors associated with death or discontinuation of the combination of atezolizumab and bevacizumab, and treatment effects, including PFS, tumor size, time to treatment failure, OS, adverse events, and tumor size, were retrospectively analyzed. HCC from hepatitis B surface antigen-susceptible patients was diagnosed as hepatitis B virus-derived HCC, and that from hepatitis C virus antibody-positive patients was diagnosed as hepatitis C-derived liver cancer.

### 2.2. Liver reserve assessment

Liver reserve was assessed using Child–Pugh scores^[7]^ and albumin-bilirubin (ALBI) grade, the latter of which was calculated using albumin and bilirubin as follows^[8]^: (ALBI score = [log10 bilirubin [µmol/L] × 0.66] + [albumin (g/L] × −0.085]). ALBI grade was defined as follows: ≤−2.60, ALBI grade 1; >−2.60, ≤−1.39, ALBI grade 2; and >−1.39, ALBI grade 3. To further subdivide moderate liver damage, mALBI grades were classified as follows: ≤−2.60, ALBI grade 1; >−2.60 to ≤−2.270, ALBI grade 2a; >−2.270 to ≤−1.39, ALBI grade 2b; and >−1.39, ALBI grade 3.^[9,10]^

### 2.3. Diagnosis of HCC

HCC was diagnosed on the basis of dynamic computed tomography, magnetic resonance imaging, and/or pathological findings. Tumor-node-metastasis staging of liver cancer by the Liver Cancer Study Group of Japan (6th edition)^[11]^ was used to evaluate tumor progression in parallel with Barcelona Clinic Liver Cancer (BCLC) staging.^[12]^

### 2.4. Determination of treatment efficacy

Antitumor efficacy was assessed using the response evaluation criteria in solid tumors (RECIST)^[13]^ and the modified RECIST^[14]^ (mRECIST). Four-phase (i.e., unenhanced, late arterial, portal, and equilibrium) contrast-enhanced computed tomography studies were performed at baseline and every 3 to 9 weeks thereafter. Cases in which contrast-enhanced imaging studies were not available were evaluated with RECIST only. Determination of treatment efficiency using mRECIST and RECIST version 1.1 was undertaken by each participating principal investigator. Hyperprogression was defined as a tumor growth kinetics ratio (TGKR) of 2 or more according to the study by Ji et al.^[15]^

### 2.5. Adverse event assessment

Adverse events associated with atezolizumab plus bevacizumab treatment were determined based on the National Cancer Institute Common Terminology Criteria for Adverse Events version 4.0. The most serious adverse events that occurred during the observation period are listed. When side effects occurred, appropriate therapeutic interventions such as discontinuation, dose reduction, and steroid administration were performed according to the guidelines for appropriate use.^[16]^

### 2.6. Statistical analyses

Data are expressed as mean and standard deviation. Statistical analyses were performed using Student t-test, Fisher exact test, Welch t-test, Cox hazard analysis, Kaplan–Meier analysis, logistic analysis, and log-rank test. Differences were considered statistically significant at a *P* value of 0.05. All statistical analyses were performed using JMP Pro version 15.1.0 (SAS Institute Inc., Cary, NC, USA). Graphs were generated using Prism version 9.1.0 (GraphPad Software, San Diego, CA, USA).

## 3. Results

**Figure 1.**
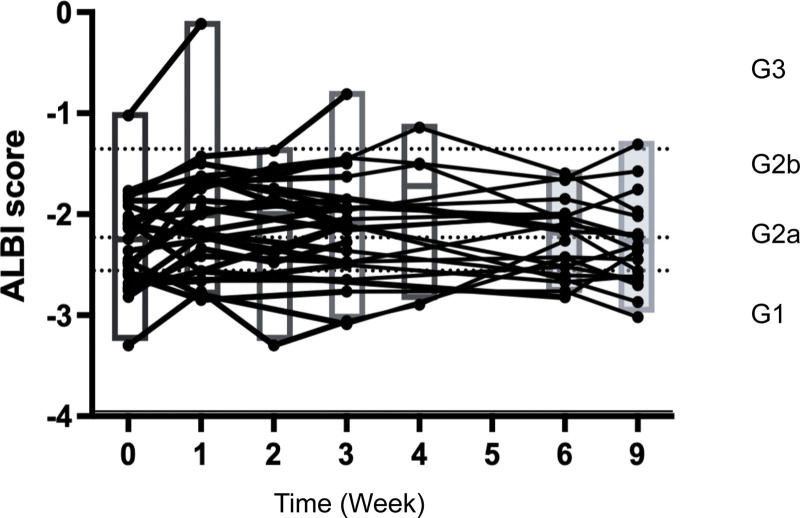
Changes in ALBI grade from the start of treatment to week 9. ALBI = albumin-bilirubin.

**Figure 2.**
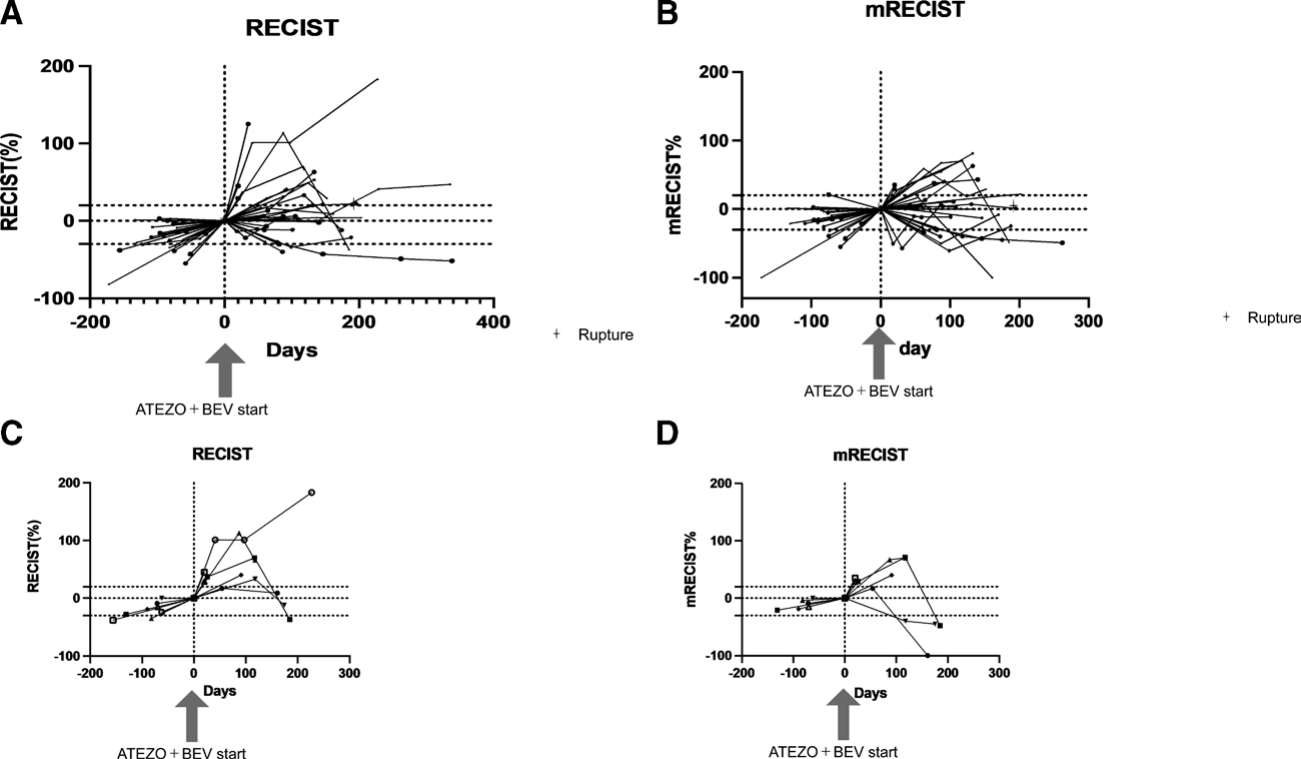
Changes in tumor size from pretreatment to the start of treatment and after the start of treatment. a. RECIST. b. mRECIST. c. Eight cases meeting the definition of hyperprogression RECIST. d. Eight cases meeting the definition of hyperprogression mRECIST.mRECIST = modified response evaluation criteria in solid tumors, RECIST = response evaluation criteria in solid tumors.

**Figure 3.**
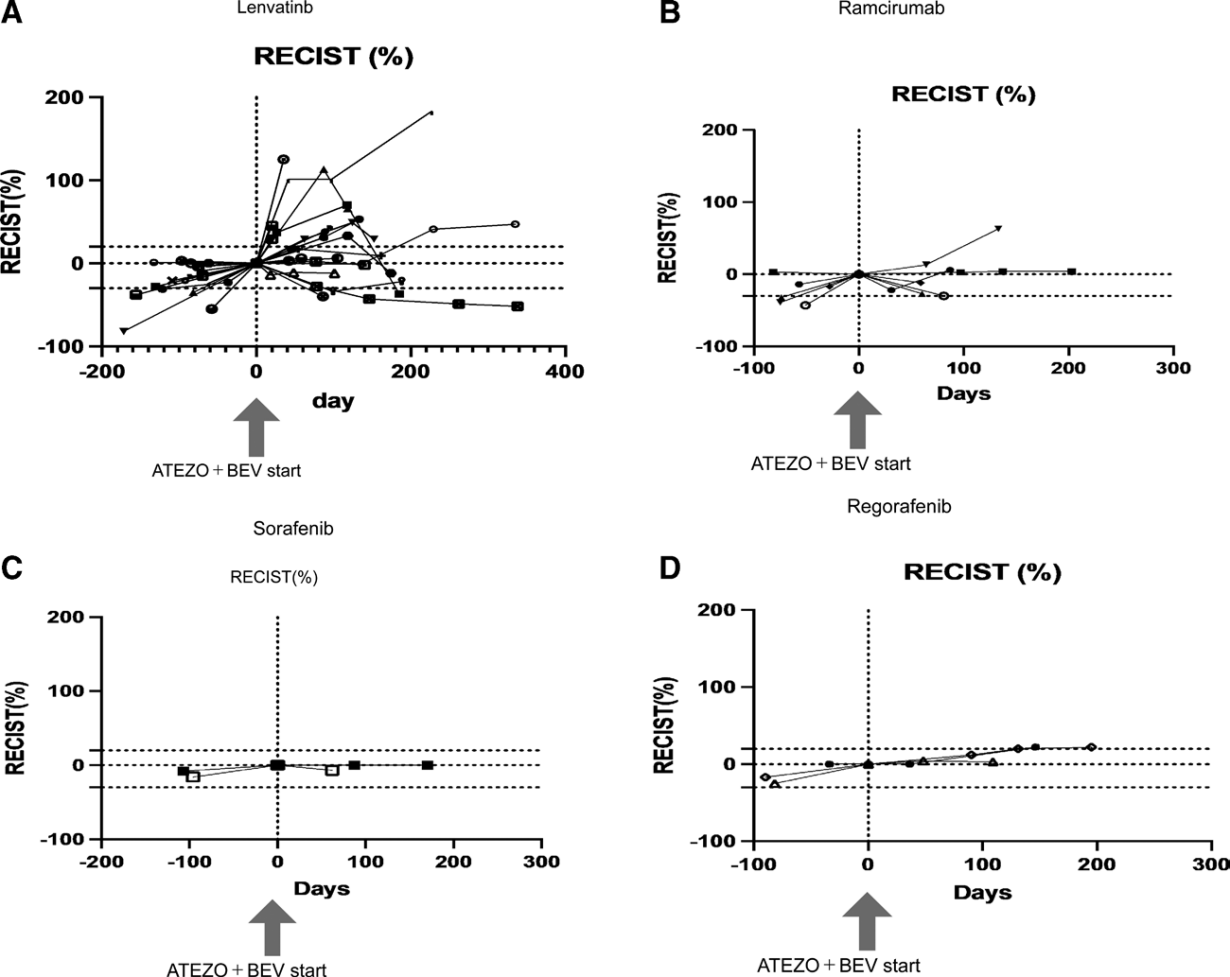
Changes in tumor size according to RECIST from pretreatment to during and after the commencement of each previous treatment. a. Lenvatinib. b. Ramucirumab. c. Sorafenib. d. Regorafenib.RECIST = response evaluation criteria in solid tumors.

**Figure 4.**
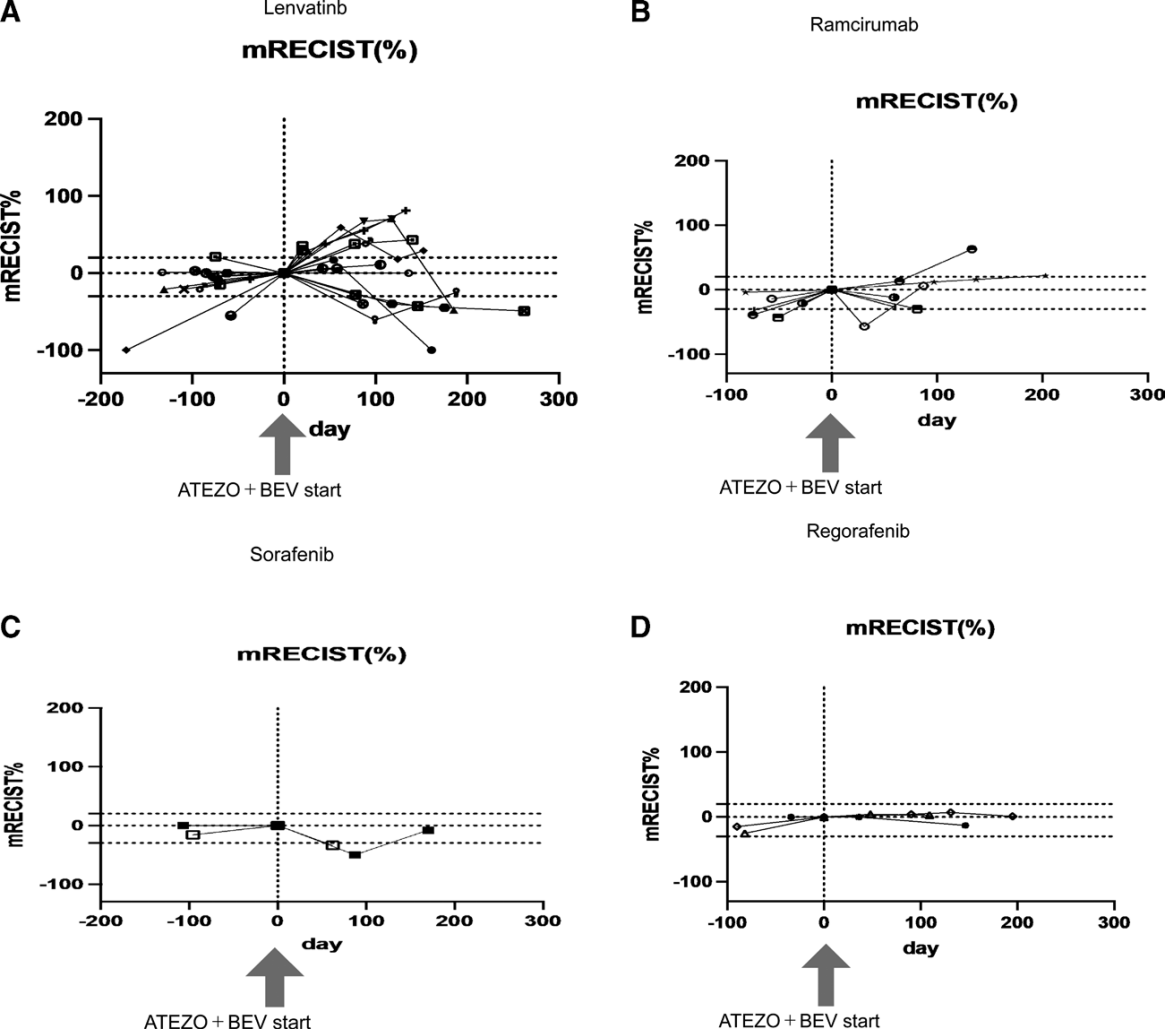
Changes in tumor size according to mRECIST from pretreatment to during and after the commencement of each previous treatment. a. Lenvatinib. b. Ramucirumab. c. Sorafenib. d. Regorafenib.mRECIST = modified response evaluation criteria in solid tumors.

**Figure 5.**
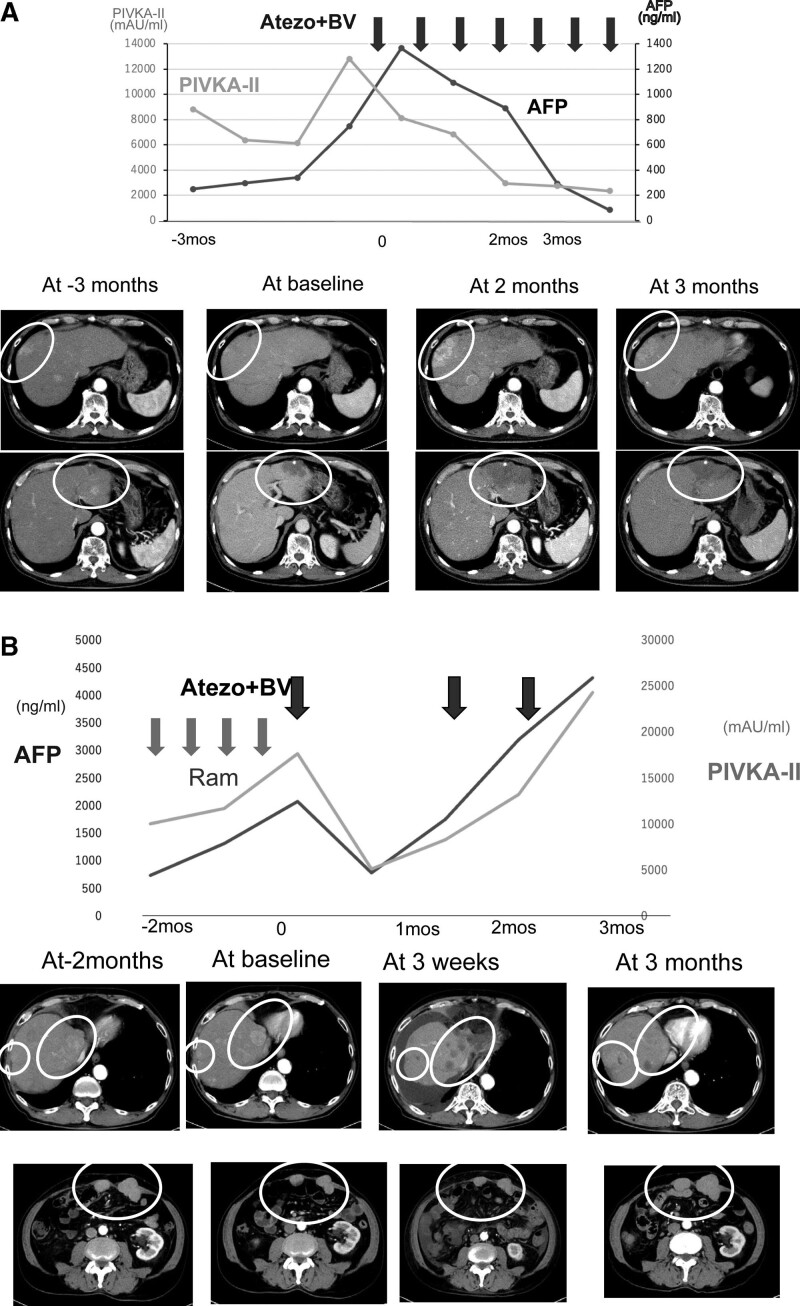
Contrast-enhanced CT images and tumor markers in characteristic cases according to previous treatment (AFP, PIVKA-II). a. Pretreatment was lenvatinib. The tumor markers decreased after the start of treatment, but tumor size according to both RECIST and mRECIST was increased. However, the size decreased with continued treatment. b. Pretreatment was ramucirumab. Early after the start of treatment, tumor size according to both RECIST and mRECIST was decreased and tumor markers were also decreased, but liver atrophy occurred and ascites appeared. The patient improved after one course of withdrawal, but tumor markers and tumor size then both increased.mRECIST = modified response evaluation criteria in solid tumors, RECIST = response evaluation criteria in solid tumors.

Thirty-one patients were enrolled in this study. A summary of background factors is provided in Table 1. Reflecting the characteristics of HCC patients in Japan, there were more aged patients than in the clinical trial (IMbrave150 study). The etiology was viral in 15 cases and non-viral in 16 cases. The Child–Pugh score was Child–Pugh A in all patients in IMbrave150, but four patients with Child–Pugh B (7 and 8 points) were included in this study, reflecting real clinical practice. The tumor status was BCLC B in 15 cases and BCLC C in 16 cases. The median treatment line was two and ranged from second to sixth line. Immediate prior treatment was lenvatinib in 20 patients, sorafenib in 2, regorafenib in 3, and ramucirumab in 6. The respective overall response rate (ORR) and disease control rate (DCR) are shown in Table 1. Side effects and tumor progression were the reasons for switching from previous therapy to atezolizumab plus bevacizumab in 23% and 73% of patients, respectively. There was no significant change in ALBI grade during the treatment period from the start to the third course (week 9) of atezolizumab + bevacizumab treatment (*P* = .3354; multivariate ANOVA) (Fig. 1). Tumor size changes are shown in spider plots in Figure 2a,b. Four of 31 patients had a partial response, 16 had no change, and 10 had tumor growth according to RECIST. In one case, the treatment was discontinued before its efficacy was determined, and thus the efficacy of the treatment could not be determined by imaging. Ten patients had a partial response, 9 had stable disease (SD), and 9 had progressive disease according to mRECIST. mRECIST could not be determined in 3 patients because imaging studies with contrast could not be performed. Among the 25 cases in which we were able to measure the pretreatment growth rate and compare it with the post-treatment rate, 8 cases had a tumor growth rate greater than or equal to 2, which met the definition of hyperprogression.^[15]^ Spider plots of RECIST and mRECIST for the 8 cases that met the definition of hyperprogression are shown in Figure 2c,d. Lenvatinib was used as pretreatment in all 8 cases. Four of these patients discontinued treatment immediately, while 4 patients continued treatment because their tumor marker levels were decreasing or their performance status improved. In 3 cases, the tumor subsequently shrank, and in 1 case, the tumor continued to grow. Analysis of spider plots by pretreatment showed that the treatment effect of atezolizumab plus bevacizumab tended to differ for each pretreatment (RECIST; Fig. 3a,b,c,d) (mRECIST; Fig. 4a,b,c,d). In previously treated lenvatinib cases, 5 out of 20 patients had rapid enlargement followed by shrinkage; 4 out of 5 of these met the definition of hyperprogression at the time of enlargement. In the case of atezolizumab plus bevacizumab after treatment with lenvatinib, there was a rapid increase in tumor size, followed by a decrease. However, for atezolizumab plus bevacizumab after treatment with regorafenib and sorafenib, all cases were almost SD. In patients treated with atezolizumab plus bevacizumab after ramucirumab treatment, there was a rapid decrease in tumor size followed by an increase. A case treated with atezolizumab plus bevacizumab after lenvatinib treatment, once clearly enlarged on imaging and then reduced, is shown in Figure 5a. The tumor markers decreased after the start of treatment, but the tumor size according to both RECIST and mRECIST increased. However, the tumor size decreased with continued treatment. None of these changes were observed in patients who had received prior treatment other than lenvatinib. The cases shown in Figure 5b treated with atezolizumab and bevacizumab after ramucirumab followed a very different course than those after lenvatinib. In these, the tumor diameter of both RECIST and mRECIST decreased early after the start of treatment and tumor markers decreased, but liver atrophy occurred and ascites appeared. The disease improved after one course of withdrawal, but both tumor markers and tumor diameter increased after the second course of treatment.

**Table 1:** Characteristics of 31 patients who received atezolizumab plus bevacizumab for hepatocellular carcinoma after MTA treatment.

**Cases**	**n = 31**
**Median age (interquartile range (IQR**))	**72 (45–88**)
**Sex** **M/F (%**)	**26 (84%)/5 (16%**)
**Etiology (HBV/HCV/NASH/ASH**)	**4 (13%)/11 (35%)/9 (29%)/7 (23%**)
**mALBI grade (1/2a/2b/3) (%**)	**7 (23%)/8 (26%)/15 (48%)/1 (3%**)
**Child–Pugh (5/6/7/8) (score**)	**17 (55%)/10 (32%)/3 (10%)/1 (3%**)
**BCLC** **B/C (%**)	**15 (48%)/16 (52%**)
**Up to 7 in/out (%**)	**5 (33%)/10 (67%**)
**Treatment line (%**) **(2/3/4/5/6**)	**17 (55%)/7 (22%)/5 (17%)/1 (3%)/1 (3%**)
**Time from initial TKI (m) (IQR**)	**18 (13–22**)
**Alpha-fetoprotein ≥ 400ng/mL (%**)	**14 (47**)
**Presence of macrovascular invasion (%**)	4 (13)
**Presence of extrahepatic spread (%**)	**12 (39**)
Pretreatment n (ORR/DCR of pretreatment)	**Len: 20 (30%/80%**) **Sor: 2(0%/50%**) **Reg: 3 (0%/33%**) **Ram: 6 (0%/16%**)
Reasons for switching from previous treatment (AE/PD)(%)	**7 (23%)/24 (77%**)

Data are expressed as median (first-third quartiles) or number (%).

Baseline data was determined at the time of atezolizumab plus bevacizumab initiation.

AE = adverse event, ASH = alcoholic steatohepatitis, BCLC = Barcelona Clinic Liver Cancer, DCR = disease control rate, HBV = hepatitis B virus, HCV = hepatitis C virus, NASH = nonalcoholic steatohepatitis, PD = progressive disease, ORR = overall response rate, TKI = tyrosine kinase inhibitor.

In a comparison of lenvatinib with other MTA groups by previous treatment, tumors grew significantly faster immediately after starting atezolizumab plus bevacizumab in the group whose previous treatment was lenvatinib (*P* = .0369; RECIST). The tumor growth kinetics rate compared with the growth rate before the start of atezolizumab plus bevacizumab treatment was also significantly greater in the lenvatinib group (*P* = .0126; mRECIST). Cases of enlargement and then shrinkage were only present in those with lenvatinib as previous treatment (*P* = .0269; *χ*^2^ test) (Table 2). No differences in PFS, OS, ORR, or DCR were observed between the different previous treatments (Table 2). ORR and DCR for all patients were 17%/60%, approximately 30%/77% of the IMbrave150 trial. The median OS for all patients was 11.4 months (95% CI 6.3–17.3) and the median PFS was 3.5 months (95% CI 1.8–4.5 months), shorter than the PFS of 6.8 months (95% CI 5.7–8.3) and OS of 19.2 months (95% CI 17.0–23.7) in the IMbrave 150 trial. In one case, the tumor was extremely large (28 cm) from the outset, rapidly grew before treatment, and ruptured on day 7 of treatment.

**Table 2:** Comparison of treatment efficacy of atezolizumab plus bevacizumab in pretreated lenvatinib and pretreated non-lenvatinib groups.

	Pretreatment with lenvatinib (n = 20)	Pretreatment with other than lenvatinib (n = 11)	*P* value
Age	70.4 ± 2.05	72.0 ± 3.30	.6524
Sex (M/F)	17/3	9/2	.8177
MVI (yes/no)	2/18	2/9	.3441
Etiology (NASH/nonNASH)	5/15	3/8	.8902
ALBI gGrade (1/2/3)	4/16/0	3/8/0	.6431
Early tumor growth kinetics (RECIST)	0.00625 ± 0.00205	−0.00128 ± 0.00438	.0369*
Early Tumor growth kinetics (mRECIST)	0.00229 ± 0.0021	−0.00322 ± 0.00269	.1181
Early Tumor growth kinetics ratio (RECIST)	2.1482 ± 0.9418	−0.4389 ± 1.1358	.0918
Early Tumor growth kinetics ratio (mRECIST)	5.2945 ± 1.4975	−1.1377 ± 1.7560	.0126*
Reduction after tumor enlargement yes/no	5/15	0/11	.0269*
ORR (RECIST)	21.0%	9.09%	.3787
DCR (RECIST)	63.1%	54.5%	.6431
PFS (months)	3.6 (1.3–6.3)	2.7 (1.3–4.4)	.9448
OS (months)	11.6 (4.7–)	11.4 (5.7–)	.9222

ALBI = albumin-bilirubin, DCR = disease control rate, MVI = macrovascular invasion, mRECIST = modified response evaluation criteria in solid tumors, NASH = nonalcoholic steatohepatitis, ORR = overall response rate, OS = overall survival, PFS = progression-free survival, RECIST = response evaluation criteria in solid tumors.

*Significant difference.

Treatment-related side effects were observed in several patients (Table 3). In the untreated IMbrave150 study, hypertension, fatigue, urinary protein, elevated transaminases, itching, and anorexia were frequently reported as side effects (7), but in this study, hypertension, anorexia, and abdominal pain were less common. Conversely, ascites, encephalopathy, rupture of liver cancer, and other symptoms associated with liver function and liver cancer progression were not reported in the clinical trials, but were relatively frequent in our second-line and later treatments. Two patients with large tumors before treatment initiation had HCC rupture immediately after treatment and at 5.5 months. The detection frequency of urinary proteins was also high. Of the 31 patients, 22 discontinued the treatment. The reasons for discontinuation were as follows: progressive disease in 11 cases (50%); liver cancer rupture in 2 cases; liver failure, ascites, and jaundice in 3 cases; liver abscess in 1 case; malaise in 1 case; myocardial infarction in 1 case; chest pain in 1 case; and Stevens–Johnson syndrome in 1 case. Of the 16 patients who were followed up after discontinuation, 9 (56%) were able to continue treatment for liver cancer.

**Table 3:** Adverse events of atezolizumab plus bevacizumab treatment.

n(%)	Links (n=31)	
	Any grade	Grade 3 or 4
Hypertension	4 (12.9)	0
Fatigue	2 (6.4)	0
Proteinuria	11 (35.4)	7 (22.5)
AST increase	7 (22.5)	5 (16.1)
Pruritus	0	
Diarrhea	0	0
Decreased appetite	1 (3.2)	0
Pyrexia	2(6.4)	0
AL T increase	4 (12.9)	3 (9.6)
Constipation	0	0
Blood bilirubin increase	4 (12.9)	2 (6.4)
Rash	0	0
Abdominal pain	1 (3.2)	0
Weight decrease	0	0
Asthenia	1 (3.2)	0
Infusion reaction	0	0
HFS	2 (6.4)	0
HCC rupture	2 (6.4)	2 (6.4)
Hepatic encephalopathy	2 (6.4)	1 (3.2)
Ascites	3 (9.6)	2 (6.4)
Stevens–Johnson syndrome	1 (3.2)	1 (3.2)
Stomatitis	1 (3.2)	0
Adrenal insufficiency	1 (3.2)	0

Data are expressed as number (%).

ALT = alanine transaminase, HCC = hepatocellular carcinoma, HFS = hand-foot syndrome.

## 4. Discussion

Sorafenib, lenvatinib, regorafenib, ramucirumab, and cabozantinib^[1–5]^ have been approved as molecular targeted therapies for unresectable advanced HCC. These inhibitors have considerably prolonged the prognosis of patients with advanced HCC, and appropriate sequential treatment methods are being explored. Atezolizumab plus bevacizumab is the first combination therapy comprising an immune checkpoint inhibitor and a molecular targeted drug to show superiority over sorafenib^[6]^ in terms of OS and PFS in the IMbrave150 trial. However, this study was conducted in patients who had not received prior therapy. Many patients with HCC are treated with MTAs, but the efficacy and safety of these regimens have not been demonstrated in such patients. In this study, we evaluated 31 patients treated with atezolizumab plus bevacizumab for advanced HCC who had previously been treated with MTAs. Both efficacy and PFS were not as good as those reported in the IMbrave150 trial, suggesting that the efficacy of second-line treatment may be worse than that of first-line treatment. Recently, the concept of hyperprogression associated with immuno-oncology therapy has been proposed.^[16,17]^ In the present case, 8 of 25 cases met the definition of hyperprogression^[18]^ at the first imaging examination within 3 months after the start of immuno-oncology therapy. In all cases, lenvatinib was administered immediately before switching. Four of the 8 patients continued treatment because their tumor markers had decreased and their side effects were mild. As a result, in 3 out of 4 cases, the tumors had shrunk at the time of imaging studies 1 to 5 months later. This rapid enlargement and shrinkage were observed only in patients previously treated with lenvatinib. MTAs have different inhibitory effects, with lenvatinib exhibiting strong antitumor activity by specifically inhibiting fibroblast growth factor (FGF) receptor (FGFR), vascular endothelial growth factor (VEGF) receptor (VEGFR), platelet-derived growth factor receptor (PDGFR), KIT, and rearranged during transfection (RET).^[19]^ In contrast, bevacizumab is a monoclonal antibody against VEGF that has no inhibitory effects on FGFR or PDGFR.^[20]^ In the final analysis of the REFLECT study, serum FGF19 and FGF23 levels increased after lenvatinib treatment.^[21]^ Furthermore, in another study, FGF19 at week 4 and FGF23 at week 8 of HCC treatment with lenvatinib were highly elevated, which was associated with treatment response.^[22]^ One study also reported that FGF23 was elevated in thyroid cancer treated with lenvatinib, which was associated with a long-term treatment response.^[23]^ Therefore, it has been suggested that the use of molecular targeted drugs that cannot suppress the FGF pathway after lenvatinib treatment may have poor antitumor effects. In addition, there are reports of poor real-world outcomes of ramucirumab after lenvatinib treatment compared with trials conducted after sorafenib treatment and poor outcomes of sorafenib after lenvatinib in terms of both OS and PFS.^[24,25]^ This transient acute exacerbation may have been a response to lenvatinib discontinuation. Hyperprogression caused by immune checkpoint inhibitors (ICIs) has been reported in many cases, and its pathogenesis is not clear^[26]^; however, it was linked to tumor-associated macrophage reprogramming following treatment of non-small cell lung cancer with ICIs.^[27]^ Hyperprogression is associated with extremely poor prognosis and rapid death.^[16–18,27,28]^ However, these incidences were all caused by ICI monotherapy or ICI combination therapy, and none of them resulted from treatment with ICI plus an MTA. There are no reports of hyperprogression followed by shrinkage. Furthermore, none of the spider plots reported in the phase 1 IMbrave150 study, which was pretreatment-free atezolizumab plus bevacizumab treatment, shrank once increased, as reported in the present study.^[29]^ It is possible that this does not reflect true hyperprogression, considering that it takes a certain period for the effect of ICIs to appear. We believe that judgment of treatment efficacy should be made carefully when atezolizumab plus bevacizumab treatment is administered after second-line treatment. However, there are also cases of rapid growth, and caution should be exercised when the tumor is too large. In contrast, all patients who received atezolizumab plus bevacizumab treatment after sorafenib and regorafenib treatment had SD. Sorafenib is a multi-kinase inhibitor with no FGFR inhibitory activity, including FGFR and tyrosine kinase with immunoglobulin and epidermal growth factor homology domains 2 (TIE-2).^[30]^ The inhibitory effect of regorafenib is weaker than that of lenvatinib.^[31]^ One study reported no increase in FGF19 and FGF23 after sorafenib treatment.^[21]^ The fact that all patients had SD after switching to atezolizumab plus bevacizumab in our analysis may be because sorafenib and regorafenib do not promote FGF19 or FGF23 overexpression. One study also reported that regorafenib enhances antitumor immunity by promoting macrophage recruitment through the inhibition of TIE2.^[32]^ This may have led to the relatively rapid activation of antitumor immunity after the switch. In some cases, patients who received atezolizumab plus bevacizumab after ramucirumab treatment showed rapid shrinkage. Ramucirumab is an antibody drug against VEGFR,^[5]^ and the subsequent administration of bevacizumab, which inhibits VEGF, may have caused rapid blood flow inhibition.

Liver reserves improved with drug withdrawal, although there were cases of temporary deterioration. After the improvement, the reserve was maintained, and it was thought that safe treatment was possible even after second-line treatment, as described in previous report.^[33]^

There were many side effects that differed from those observed during the first-line treatment. The relatively low incidence of side effects such as hypertension and fatigue was likely due to the fact that the side effects had already been controlled by previous MTA treatment. However, side effects related to liver reserve, such as ascites and hepatic encephalopathy, which were not observed in the IMbrave150 study, were relatively common. In some cases, ascites jaundice appeared after the start of treatment, even though the Child–Pugh score at the start of atezolizumab plus bevacizumab was 5 points. All patients with a history of urinary protein or ascites during previous treatment relapsed. It is known that liver reserve gradually declines with MTA treatment.^[34]^ In these cases, although the liver reserve seemed to have improved at the start of atezolizumab plus bevacizumab treatment, it is possible that the reserve declined. Regarding urinary proteins, it is thought that VEGF inhibition by MTAs in the previous treatment caused thinning of endothelial cells^[35]^ and led to the occurrence of urinary proteins more frequently than in the first-line treatment.

This study had some limitations, including the fact that it was an observational study without allocation. In addition, too few cases were analyzed to obtain definitive conclusions. Furthermore, the observation period was short; thus, the long-term prognosis and side effects are not yet known. Nevertheless, the present study suggests that the course of atezolizumab plus bevacizumab treatment after the second line differs from the first line and may depend on the previous treatment.

## 5. Conclusions

The results of atezolizumab plus bevacizumab treatment for HCC after MTA treatment were reported. The effect of atezolizumab plus bevacizumab treatment depended on prior therapy, with cases showing rapid increases in tumor size and then shrinkage when the prior therapy was lenvatinib. Side effects were mostly related to liver function, unlike the previously reported atezolizumab plus bevacizumab as first-line treatment.

## Acknowledgements

We thank H. Nikki March, PhD, from Edanz (https://jp.edanz.com/ac) for editing this manuscript.

## Author contributions

RS participated in the design of this study, measured and analyzed RECIST and mRECIST, performed the formal analysis, and drafted the manuscript. TS and AU participated in data curation and review. TS and YT participated in conceptualization and data curation. SY, TK, TK, NH, TN, and MT participated in data curation and measured and analyzed RECIST and mRECIST. YA, AO, MO, and TM participated in data curation and measured and analyzed RECIST and mRECIST. KU, MK, ST, YA, TY, NY, and SH participated in data curation. MN participated in the study. KM and MK participated in the conceptualization and review.

**Conceptualization:** Rie Sugimoto, Takeshi Senju, Yuki Tanaka, Kenta Motomura, Motoyuki Kohjima.

**Data curation:** Rie Sugimoto, Takeaki Satoh, Akihiro Ueda, Takeshi Senju, Yuki Tanaka, Shinsaku Yamashita, Toshimasa Koyanagi, Tomoyuki Kurashige, Nobito Higuchi, Tsukasa Nakamura, Masatake Tanaka, Yuuki Azuma, Akari Ohno, Aritsune Ooho, Mari Ooe, Taiji Mutsuki, Koutarou Uchimura, Masami Kuniyoshi, Seiya Tada, Yoshifusa Aratake, Tsuyoshi Yoshimoto, Naoki Yamashita, Shigeru Harada.

**Formal analysis:** Rie Sugimoto.

**Funding acquisition:** Rie Sugimoto.

**Investigation:** Rie Sugimoto, Shinsaku Yamashita, Toshimasa Koyanagi, Tomoyuki Kurashige, Nobito Higuchi, Tsukasa Nakamura, Masatake Tanaka, Yuuki Azuma, Akari Ohno.

**Methodology:** Rie Sugimoto.

**Project administration:** Rie Sugimoto.

**Visualization:** Rie Sugimoto.

**Writing – original draft:** Rie Sugimoto.

**Writing – review & editing:** Takeaki Satoh, Akihiro Ueda, Makoto Nakamuta, Kenta Motomura, Motoyuki Kohjima.
